# Specificity and complexity in bacterial quorum-sensing systems

**DOI:** 10.1093/femsre/fuw014

**Published:** 2016-06-26

**Authors:** Lisa A. Hawver, Sarah A. Jung, Wai-Leung Ng

**Affiliations:** 1 Department of Molecular Biology and Microbiology, Tufts University School of Medicine, Boston, MA 02111, USA; 2 Program in Molecular Microbiology, Sackler School of Graduate Biomedical Sciences, Tufts University, Boston, MA 02111, USA

**Keywords:** intercellular communication, gene expression, group behavior, regulatory network, chemical signaling

## Abstract

Quorum sensing (QS) is a microbial cell-to-cell communication process that relies on the production and detection of chemical signals called autoinducers (AIs) to monitor cell density and species complexity in the population. QS allows bacteria to behave as a cohesive group and coordinate collective behaviors. While most QS receptors display high specificity to their AI ligands, others are quite promiscuous in signal detection. How do specific QS receptors respond to their cognate signals with high fidelity? Why do some receptors maintain low signal recognition specificity? In addition, many QS systems are composed of multiple intersecting signaling pathways: what are the benefits of preserving such a complex signaling network when a simple linear ‘one-to-one’ regulatory pathway seems sufficient to monitor cell density? Here, we will discuss different molecular mechanisms employed by various QS systems that ensure productive and specific QS responses. Moreover, the network architectures of some well-characterized QS circuits will be reviewed to understand how the wiring of different regulatory components achieves different biological goals.

## Abstract (precis)

This review focuses on the specificity and complexity of quorum-sensing circuits in both Gram-negative and Gram-positive bacterial species.

## INTRODUCTION

### Quorum sensing

Bacterial quorum sensing (QS) is a cell-to-cell communication process that relies on the production, secretion and detection of autoinducer (AI) signals to regulate gene expression in response to changes in population density. QS allows a group of bacterial cells to regulate their gene expression in unison, which is important for carrying out group behaviors such as bioluminescence production, biofilm formation, genetic exchange and virulence factor expression (Ng and Bassler 2009). Bacterial species depend on QS to regulate important cellular processes that are essential for surveillance, survival and adaptation to their changing environments (Bassler and Vogel 2013). By monitoring the accumulation of specific AIs, bacteria can track shifts in population density and species complexity in the vicinity and respond as a group accordingly (Fuqua and Greenberg 2002; Pappas, Weingart and Winans 2004; Novick and Geisinger 2008; Ng and Bassler 2009; Williams and Camara 2009; Ng *et al*. 2011).

**Figure 1.**
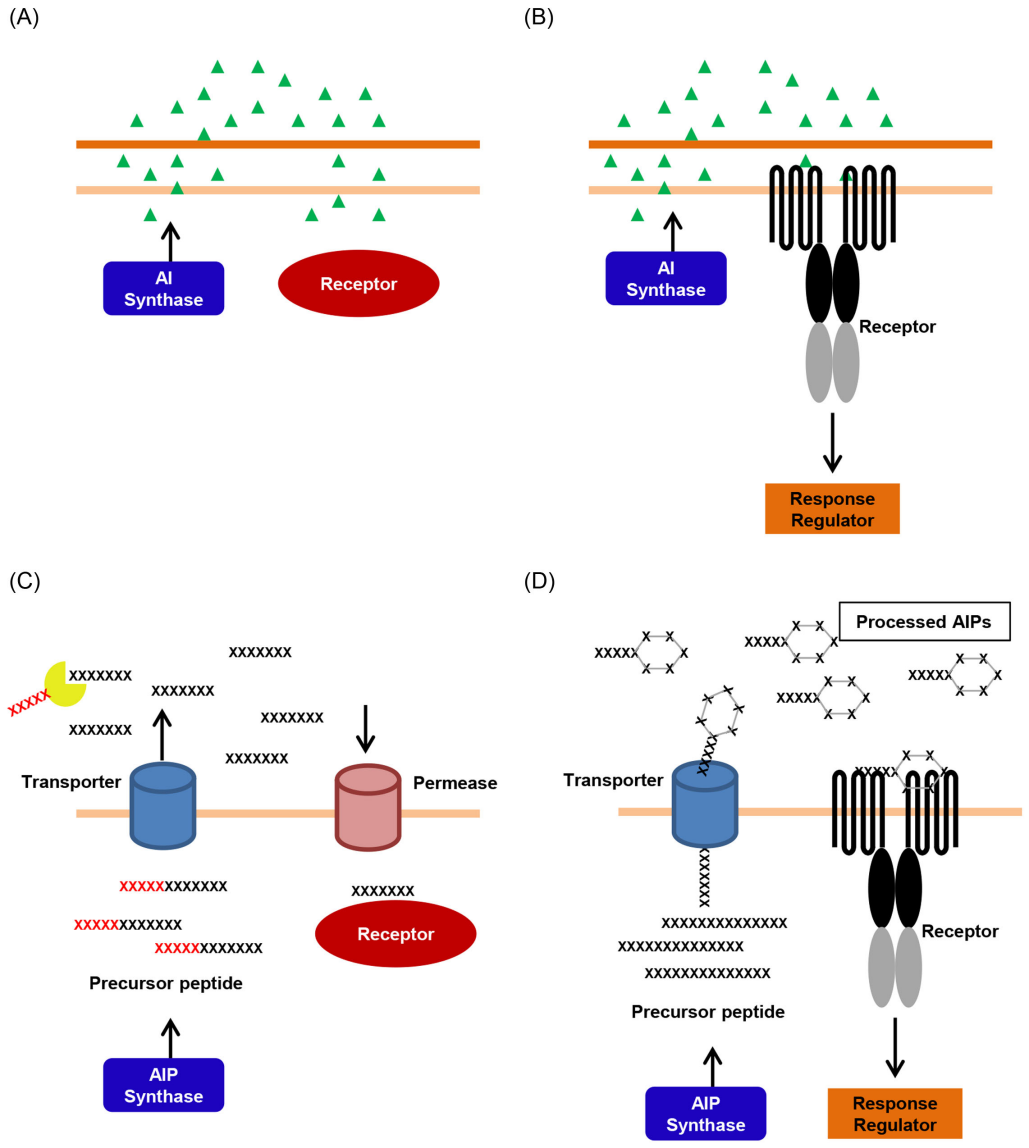
Basic QS circuit diagrams. (**A**) A Gram-negative one-component QS system. Autoinducer molecules are produced by the AI synthase, released into the extracellular environment, which are then diffused back into the cytoplasm where the QS receptor detects them, while also acting as a transcriptional regulator. (**B**) A Gram-negative two-component QS system. Autoinducer molecules are produced by the AI synthase, released into the extracellular environment, and are then detected by a transmembrane receptor. Detection of autoinducers triggers a phospho-relay that controls the downstream QS response. (**C**) A Gram-positive one-component QS system. Autoinducer peptides are produced by the AIP synthase and then released into the extracellular environment through a transporter, where they undergo proteolysis and are then transported back into the cytoplasm through a permease. In the cytoplasm, the modified AIP is detected by a QS receptor that also acts as a transcriptional regulator. (**D**) A Gram-positive two-component QS system. Autoinducer peptides are produced by the AIP synthase and released into the extracellular environment through a transporter where they undergo post-translational modifications and are then detected by a transmembrane receptor. Detection of autoinducer triggers a phospho-relay that controls the downstream QS response.

**Figure 2.**
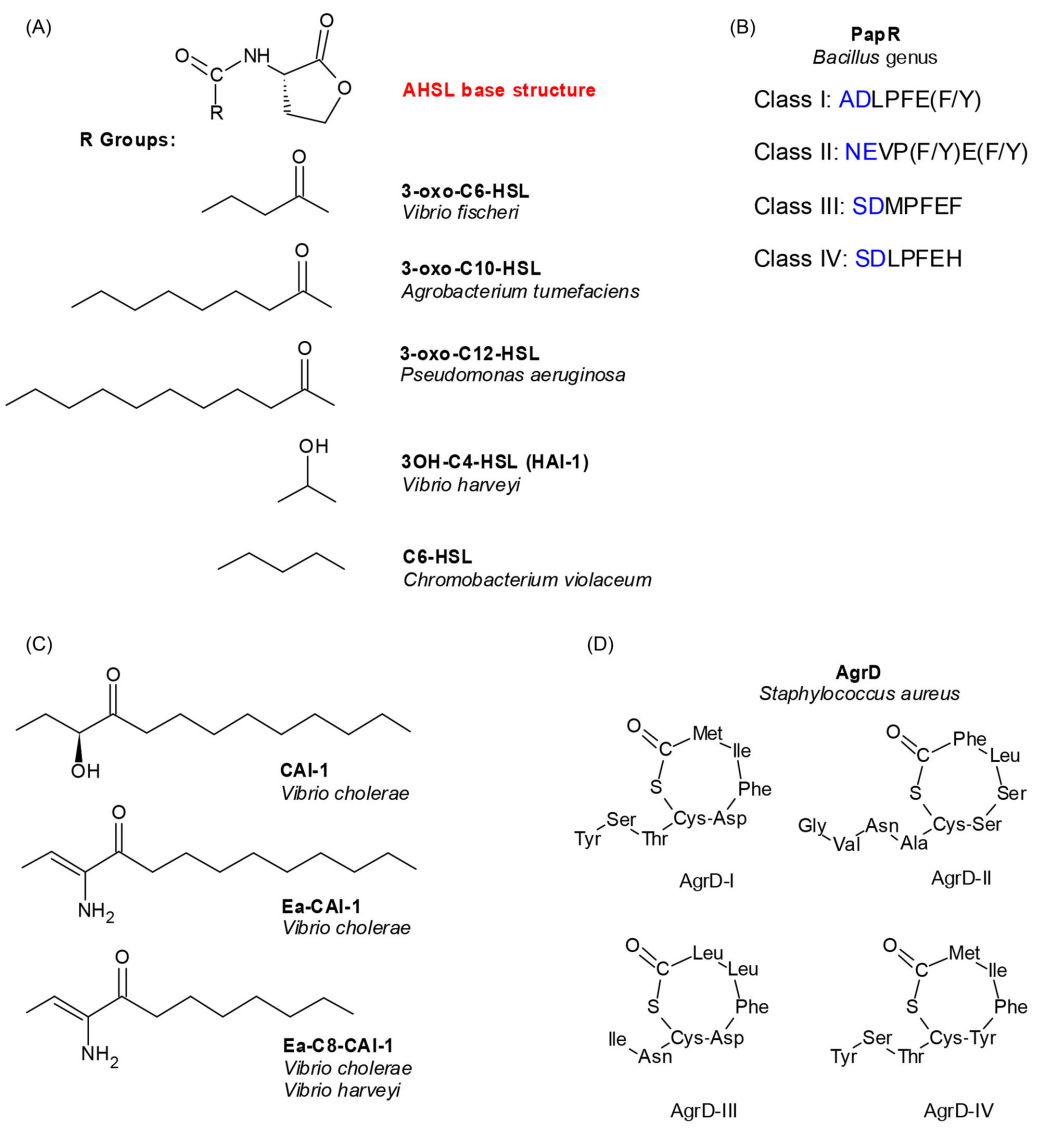
Structures of bacterial autoinducers. (**A**) Acyl-homoserine lactones (AHSLs) that are produced by various Gram-negative bacteria. Shown is the AHL base structure, plus various R groups that differ among species. (**B**) Small peptide autoinducers (AIPs) called PapR produced by *Bacillus* genus. Predicted physiologically-relevant heptapeptides are indicated by additional residues in blue. (**C**) CAI-1 and its related autoinducers produced by *Vibrio* species. (**D**) The four AgrD variants produced by *Staphylococcus aureus*.

QS systems have been identified in both Gram-negative and Gram-positive bacterial species. After synthesis, the signal is exported extracellularly, and its concentration increases proportionally to population density. When the concentration of the signal is above a certain threshold, the signal is detected by a QS receptor that elicits a downstream signal transduction cascade, triggering a high cell density gene expression program (Fig. 1). In Gram-negative bacteria, AI molecules are comprised of several chemical classes including acyl homoserine lactones (AHSLs), alkylquinolones, α-hydroxyketones and diffusible signal factor (fatty acid-like compounds) (Fig. 2). These signaling molecules are synthesized from common metabolites such as fatty acids, anthranilate and *S*-adenosylmethionine (SAM), either with a single signal synthase or through a series of enzymatic reactions (Fuqua and Greenberg 1998; Tiaden, Spirig and Hilbi 2010; Ryan *et al*. 2015). In contrast to the signals that regulate QS in Gram-negative bacteria, short oligopeptides are produced and detected in Gram-positive bacteria (Fig. 2). In most cases, the signal is synthesized as a longer peptide precursor, which is subsequently exported and modified upon its secretion by a dedicated transporter (Fig. 1C and D) (Kleerebezem *et al*. 1997; Pottathil and Lazazzera 2003; Lyon and Novick 2004; Dufour and Levesque 2013; Cook and Federle 2014; Monnet, Juillard and Gardan 2014).

In general, a specific set of AIs is produced and detected by each QS bacterial species; nonetheless, it is not unreasonable to assume that these species are exposed to non-cognate signal analogs produced by other species in the vicinity. How does a QS receptor detect and distinguish related AIs with similar structures? How do bacteria prevent premature induction of QS caused by signal fluctuations? Moreover, bacterial QS systems have likely evolved independently of one another, yet, there are elegant patterns within these circuits regarding signal detection and system architecture. Here, we will review how bacteria ensure steadfast QS signal transduction that only occurs upon detection of cognate signals with corresponding receptors. Specifically, we will discuss how several types of QS receptors have evolved to possess an exquisite and intrinsic specificity towards their cognate ligands. We will then consider strategies that maintain signal transduction specificity and fidelity in various QS systems and why multiple and distinct regulatory pathways are connected to form a complex signaling network.

## LIGAND SPECIFICITY IN QS RECEPTORS

### LuxR-type regulators

QS was first discovered as a regulatory mechanism of bioluminescence induction in the Gram-negative bacterium *Vibrio fischeri*, where light production is induced through a cell-density dependent activation of expression of the luciferase operon (Nealson, Platt and Hastings 1970; Nealson and Hastings 1979). This marine bacterium employs a LuxI-LuxR-type QS system to control light production, in which LuxR serves as both the cytoplasmic AI receptor and the transcriptional activator of the *lux* operon (Fig. 1A) (Engebrecht, Nealson and Silverman 1983; Engebrecht and Silverman 1984; Stevens, Dolan and Greenberg 1994; Schaefer *et al*. 1996a; Stevens and Greenberg 1997; Stevens *et al*. 1999). LuxI is the AI synthase, which catalyzes the production of *N*-(3-oxohexanoyl)-homoserine lactone (3-oxo-C6-HSL), the cognate signal for *V. fischeri* LuxR (Fig. 2A) (Eberhard *et al*. 1981; Engebrecht and Silverman 1984; Schaefer *et al*. 1996b).

LuxR and other similar regulators in the family are composed of two distinct domains: an N-terminal ligand binding domain (LBD) and a C-terminal DNA-binding domain (DBD) (Shadel, Young and Baldwin 1990; Slock *et al*. 1990; Choi and Greenberg 1991, 1992; Fuqua and Greenberg 1998). In the absence of AI, most LuxR-type proteins do not fold correctly and are rapidly degraded; however, the complex becomes stable upon ligand binding. In the *V. fischeri* LuxR, ligand (3-oxo-C6-HSL) binding also induces a conformational change that reveals the DBD of LuxR, therefore rendering it free to bind to the promoter of the *lux* operon and activate its transcription (Stevens, Dolan and Greenberg 1994; Hanzelka and Greenberg 1995). Homologs of the LuxI-LuxR QS system have been identified in many Gram-negative bacteria, including LasI-LasR (Passador *et al*. 1993; Pearson *et al*. 1994; Bottomley *et al*. 2007; Zou and Nair 2009), RhlI-RhlR (Ochsner *et al*. 1994; Brint and Ohman 1995; Ochsner and Reiser 1995; Pearson *et al*. 1995), QscR from *Pseudomonas aeruginosa* (Chugani *et al*. 2001), TraI-TraR from *Agrobacterium tumefaciens* (Fuqua and Winans 1994; Hwang *et al*. 1994; Vannini *et al*. 2002; Zhang *et al*. 2002) and CviR from *Chromobacterium violaceum* (McClean *et al*. 1997; Chen *et al*. 2011; Stauff and Bassler 2011). In these systems, AIs synthesized by the different signal synthases share a common homoserine lactone group, but differ in acyl chain length and modifications (Fig. 2A). Since these AIs are structurally similar and many QS species are capable of producing them, signal interference among non-cognate systems could lead to an unwanted, premature induction of the QS response. Thus, many QS bacteria have evolved their LuxR-type receptors with an exquisite specificity towards its cognate signal. On the other hand, some LuxR-type receptors display a more relaxed specificity and detect multiple ligands, such as CviR discussed later in this review (Swem *et al*. 2009; Chen *et al*. 2011). While the exact biological role for this promiscuous signal specificity is unknown, it is thought that the latter class of receptors can be used for inter-species signaling. Here, we use a few well-characterized LuxI-LuxR-type QS systems to illustrate the diversity in signal production and detection specificity.

### AI binding is required for protein folding of some LuxR-type regulators

*Agrobacterium tumefaciens* employs a LuxI-LuxR-type QS system, called TraI-TraR, to regulate the transfer of the Ti plasmid from the bacterium to its plant host, ultimately causing tumor formation inside the host (Piper, Beck von Bodman and Farrand 1993; Fuqua and Winans 1994; Hwang *et al*. 1994; Christie 1997). *Agrobacterium tumefaciens* produces several AHSLs but the most abundant one is 3-oxo-C10-HSL (Fig. 2A), which is synthesized by TraI and is the cognate ligand of QS receptor TraR (Hwang *et al*. 1994; Zhu *et al*. 1998). Several unrelated AHSLs are also capable of activating TraR, however, these analogs are only active when present at high concentrations (Zhu *et al*. 1998). These findings suggest that TraR has evolved a very specific interaction with its cognate signal and is refractory to being activated by non-cognate signals in physiologically-relevant conditions. The 3D structure of TraR bound with its cognate AHSL ligand was the first reported among all LuxR-type receptors (Vannini *et al*. 2002; Zhang *et al*. 2002). The resolved structural model suggests that TraR requires its cognate ligand for proper folding, a characteristic shared with some of the other members of the LuxR family (Vannini *et al*. 2002; Zhang *et al*. 2002). The TraR protein is a symmetric homodimer, with each monomer comprised of a LDB and a DBD that are joined by a short linker region. Surprisingly, the 3-oxo-C10-HSL ligand is enclosed in a solvent-inaccessible region on the opposite side of the dimerization site of each monomer. The AI binding site is composed of aromatic and hydrophobic amino acids within each LBD. 3-oxo-C10-HSL interacts with TraR between the central β-sheet and α-helices α3, α4 and α5 to ensure that the signal remains bound and correctly-oriented (Vannini *et al*. 2002; Zhang *et al*. 2002). The invariant homoserine lactone portion of 3-oxo-C10-HSL interacts with multiple residues in the LBD, conserved among the majority of the LuxR-type receptors. While each AHSL signal is built upon a homoserine lactone core, the acyl chain of the AHSLs differs among the QS systems. These differences dictate how the molecule fits into the binding pocket of each LuxR-family receptor. For example, the acyl portion of the 3-oxo-C10-HSL interacts with TraR at residues Y53, L40, Y61, F62, some of which are not conserved. The fact that the AI binds within a buried hydrophobic pocket in the LBD suggests that AI binding plays a role in the correct folding and stabilization of the TraR protein and explains why TraR displays such high specificity for 3-oxo-C10-HSL (Zhu and Winans 1999, 2001; Vannini *et al*. 2002; Zhang *et al*. 2002).

While ligand binding is essential for TraR folding and stability, some LuxR proteins, such as LasR from *P. aeruginosa* and LuxR from *V. fischeri*, bind to their cognate ligands reversibly (Urbanowski, Lostroh and Greenberg 2004; Sappington *et al*. 2011). Indeed, low levels of active recombinant LasR can be detected in *Escherichia coli* grown without 3-oxo-C12-HSL, suggesting that LasR can fold into a functional conformation in the absence of signal through an unknown mechanism; however, this ligand-free form of LasR is very unstable (Sappington *et al*. 2011). Reversible AI binding allows the receptor to be inactivated via rapid dilution of ligand, which could be critical for the bacterium to switch behaviors from those of high cell density to those of low cell density. The crystal structure of the LBD of LasR bound to its cognate AI 3-oxo-C12-HSL provided further insight into how LasR detects its cognate AIs (Bottomley *et al*. 2007). Similar to TraR, LasR is a homodimer, in which one AI molecule binds to each monomer of the receptor in a solvent-inaccessible pocket comprised of a β-sheet and several α-helices (Bottomley *et al*. 2007). Likewise, LasR interacts with 3-oxo-C12-HSL by forming multiple hydrogen bonds in between several conserved LasR residues and the invariant homoserine lactone portion of the AI (Bottomley *et al*. 2007). However, the acyl chain of 3-oxo-C12-HSL is surrounded within a hydrophobic pocket in LasR by interacting with several non-conserved residues, possibly giving LasR the ability to distinguish 3-oxo-C12-HSL from other AHSLs (Bottomley *et al*. 2007).

### Interactions of two AHSL signals in *Vibrio fischeri* LuxR

Many QS bacteria produce multiple related AHSLs using different LuxI-type synthases. For example, aside from LuxI, *V. fischeri* carries another non-homologous AHSL synthase called AinS which produces C8-HSL. Together, these two AHSLs regulate bioluminescence production (Kuo, Callahan and Dunlap 1996; Hanzelka *et al*. 1999). C8-HSL is detected by a membrane-bound receptor AinR (we will discuss this type of AHSL receptor below). The ligand specificity of *V. fischeri* LuxR is somewhat stringent as several AHSL analogs such as 3-oxo-C5-HSL, 3-oxo-C8-HSL and 5-oxo-C6-HSL, are capable of activating *lux* expression through binding to LuxR in a heterologous *E. coli* host, but none of these analogs are as effective as the cognate signal 3-oxo-C6-HSL (Schaefer *et al*. 1996a). Although C8-HSL can bind to LuxR, the C8-HSL/LuxR complex is a weaker activator of the *lux* operon than the 3-oxo-C6-HSL/LuxR complex (Kuo, Callahan and Dunlap 1996; Schaefer *et al*. 1996a; Lupp *et al*. 2003). C8-HSL and 3-oxo-C6-HSL compete for the same binding site on LuxR and, therefore, high concentrations of C8-HSL could inhibit light production. Not surprisingly, this inhibition is suppressed by high doses of 3-oxo-C6-HSL (Schaefer *et al*. 1996a). Intriguingly, production of these two AHSLs varies among different *V. fischeri* isolates; some brighter strains that produce more luciferase, such as MJ1, secrete >1000-fold 3-oxo-C6-HSL and 5-fold less C8-HSL than other dimmer isolates (Boettcher and Ruby 1995). LuxR also displays only 75% identity among these different isolates. Directed evolution of LuxR that responds to C8-HSL but not 3-oxo-C6-HSL, reveals that residues both inside and outside of the LBD are responsible for this switch in ligand specificity (Collins, Arnold and Leadbetter 2005; Collins, Leadbetter and Arnold 2006; Hawkins *et al*. 2007). How different natural LuxR variants respond to these two competing AHSLs, and how polymorphisms among different LuxR proteins affects bioluminescence production in *V. fischeri*, remains unclear. Together, however, these findings strongly suggest that LuxR has evolved to respond to different AI compositions in order to achieve various biological goals.

### Signal antagonism revealed in CviR bound with different ligands

CviR is a cytoplasmic QS receptor in *C. violaceum*, a bacterium known for the production of a purple pigment called violacein, along with biofilm formation and cyanide production (Stauff and Bassler 2011). The production of violacein is regulated by QS via CviR activation of the ‘vioABCD’ operon (McClean *et al*. 1997; August *et al*. 2000). CviR from different *C. violaceum* isolates displays various AHSL signal specificity. In strain ATCC 31532, the cognate signal for CviR is C6-HSL (Fig. 2A), which is synthesized by CviI. However, this CviR has promiscuous ligand specificity, as CviR can activate *vioA* transcription when bound to AHSLs with acyl chain lengths ranging from C4 to C8 (Swem *et al*. 2009). Although C4-HSL can induce maximum *vioA* expression, it requires a much higher signal concentration than cognate C6-HSL (Swem *et al*. 2009). In contrast, AHSLs with acyl chain lengths ranging from C10 to C14 are either weak agonists or are completely inactive. Indeed, C10-HSL is an effective CviR antagonist (Chen *et al*. 2011). Furthermore, CviR activity can also be antagonized by a synthetic molecule analogous to C6-HSL with two modifications: a phenoxy group at the end of the acyl chain and a homocysteine thiolactone ring in place of a homoserine lactone ring (Swem *et al*. 2009). Antagonism is further increased by the addition of a chlorine atom at the ‘para’ position on the phenoxy ring and by the removal of the methyl group at the ‘ortho’ position of the phenoxy ring (antagonist CTL). Additional antagonism is observed upon the replacement of sulfur in the thiolactone with an oxygen atom (antagonist CL).

When the structure of full-length CviR bound with antagonist chlorolactone (CL) was solved (Chen *et al*. 2011), it was discovered that the domain arrangement of the receptor diverges from the structure of TraR bound with its ligand. In the CviR/CL complex, the DBD is positioned underneath the LBD of the opposite monomer in a cross-domain structure. In this conformation, the DBD domains are far apart and incompatible with operator DNA binding. Moreover, bacterial two-hybrid studies showed that only C6-HSL allows CviR interaction with the RNA polymerase α-subunit (Chen *et al*. 2011).

Interestingly, residue M89 in the LBD exists in two separate orientations when different ligands are bound to CviR. This residue is typically buried when the C6-HSL agonist is bound. However, structures of the LBD alone bound to antagonists reveal that M89 acts as a gate that swings away from the binding pocket to accommodate larger HSLs such as C8-HSL, C10-HSL and CL. Activity of CviR bound to antagonist C10-HSL is restored when M89 is mutated to smaller residues (M89S or M89A), but not when M89 is mutated to residues that were similar in size or larger. CviR with mutation M89S or M89A bound to C10-HSL demonstrated an open conformation similar to CviR/C6-HSL complexes. The mutant CviR-C10-HSL could bind DNA, interact with RNA polymerase, and initiate *vioA* transcription (Chen *et al*. 2011).

Another *C. violaceum* strain (ATCC 12472) produces 3-OH-C10-HSL as its cognate AI and also responds to C10-HSL and CL, antagonists of the previously-studied CviR from another strain (ATCC31532). Interestingly, the CviR receptor from ATCC 12472 has a Ser residue at position 89, favoring a more open binding pocket that can bind C10-HSLs. However, a second amino acid change, N77Y, together with S89M, is necessary to switch ligand specificity for this CviR to sense C10-HSL and CL as antagonists (Chen *et al*. 2011). Thus, it appears that the two CviR receptors in these two C. *violaceum* strains have evolved to specifically detect the corresponding cognate AHSL signal. This series of structure-function analyses also gives important insight into how LuxR- type receptors discriminate structurally similar molecules and illustrate a possible antagonism mechanism for this important class of QS regulators.

### Orphan (solo) LuxR-type receptors

While LuxR-type receptors and LuxI synthases are usually encoded in the same operon, some LuxR-type receptors are found to be orphans (or solos), meaning they have no genetically linked cognate AHSL synthases. These QS receptors are originally thought to respond only to AHSLs, however, it was recently found that these orphan LuxR-type proteins could respond to signals unrelated to AHSLs (Brachmann *et al*. 2013; Nguyen *et al*. 2015). Unsurprisingly, residues that are responsible for making contacts with AHSLs in other LuxR-type receptors are substituted in these orphan receptors. In the case of orphan regulators PluR and PauR, some of these substituted residues are essential for cognate signal sensing but not solely responsible for ligand-binding specificity (Brameyer and Heermann 2015).

### Ligand specificity in Gram-positive RNPP-type receptors

A few years before Hastings and colleagues published their discovery of autoinduction of bioluminescence in *V. fischeri* (Nealson, Platt and Hastings 1970), a similar discovery was made in *Streptococcus pneumoniae* by Alexander Tomasz, in which the competence state of the bacterium can be induced by a hormone-like substance present in the culture medium (Tomasz 1965). Though this finding was not recognized as a QS-regulated behavior at the time, it is now known that a peptide-based QS system is involved in competence regulation (Havarstein, Coomaraswamy and Morrison 1995), and that this likely to be the first piece of evidence of cell–cell communication. In general, Gram-positive bacteria produce and detect short oligopeptides for QS regulation (Fig. 2B and D). These peptides are usually 5 to 34 amino acids in length and undergo critical modifications upon secretion into the extracellular space (Fig. 1C and D) (Kleerebezem *et al*. 1997; Pottathil and Lazazzera 2003; Lyon and Novick 2004; Dufour and Levesque 2013; Cook and Federle 2014; Monnet, Juillard and Gardan 2014). One family of this type of QS receptor is called the RNPP family, an acronym for the four receptors that are classified within this category: Rap, NprR, PlcR and PrgX (Declerck *et al*. 2007; Rocha-Estrada *et al*. 2010). For these systems, signal precursors are first synthesized as ∼40 amino acid peptides that contain a signal sequence for peptide export via either the general secretion system (Sec) or by dedicated ABC transporters (Jimenez and Federle 2014). The C-terminal region of these peptide signals contains ∼13 to 20 amino acids that are modified by proteolysis (Fig. 1C) (Pottathil and Lazazzera 2003; Declerck *et al*. 2007; Gohar *et al*. 2008; Rocha-Estrada *et al*. 2010). The modified peptides are then imported back into the cell through an oligopeptide permease for subsequent cytoplasmic receptor detection (Fig. 1C) (Perego 1997; Gominet *et al*. 2001). Rap is an aspartyl phosphate phosphatase and transcriptional activator protein (Rocha-Estrada *et al*. 2010; Parashar *et al*. 2011), while NprR, PlcR and PrgX are DNA-binding transcription factors (Wintjens and Rooman 1996; Aravind *et al*. 2005; Rocha-Estrada *et al*. 2010). NprR is a neutral protease regulator (Pottathil and Lazazzera 2003; Zouhir *et al*. 2013), PlcR is a phospholipase C regulator (Declerck *et al*. 2007; Gohar *et al*. 2008) and PrgX regulates plasmid conjugative transfer (Bae, Clerc-Bardin and Dunny 2000; Shi *et al*. 2005). Structural and mechanistic analyses of the RNPP-type receptors have been reviewed recently (Rocha-Estrada *et al*. 2010; Cook and Federle 2014). Here, we will use the well-characterized PlcR system to illustrate ligand specificity control in this family of regulators.

PlcR is a cytoplasmic QS receptor known as a phospholipase C regulator in the Gram-positive *Bacillus* genus, including *B. anthracis*, *B. cereus, B. thuringiensis*, and other *Bacillus* species (Lereclus *et al*. 1996; Declerck *et al*. 2007). The peptide ligand of PlcR is called PapR. There are four PlcR/PapR classes within the *B. cereus* group, identified as PlcRI/PapRI (LPFE(F/Y)), PlcRII/PapRII (VP(F/Y)E(F/Y)), PlcRIII/PapRIII (MPFEF) and PlcRIV/PapRIV (LPFEH) (Fig. 2B) (Slamti and Lereclus 2002, 2005). The PapR variations are generally in the first and last residues of the pentapeptide (the longer, physiologically-relevant forms of these peptides will be discussed below), giving rise to species ligand specificity (Slamti and Lereclus 2002, 2005). The X-ray crystal structure of *B. thuringiensis* PlcRI bound with PapR (LPFEF) revealed that PlcRI is a symmetrical, dimeric protein composed of an N-terminal DBD and a C-terminal LBD, which also serves as the dimerization site between the two monomers (Declerck *et al*. 2007). The LBD is comprised of five tetratricopeptide repeats (TPR) (Declerck *et al*. 2007; Grenha *et al*. 2013). TPR domains are known for their protein–protein and protein–peptide interactions (Blatch and Lassle 1999) and the two PlcR monomers meet at their respective TPR domains which also contain the PapR-binding sites. Within this binding site, the backbone of the PapR ligand forms hydrogen bonds with several residues of PlcRI. Specially, the PapR glutamic acid binds to Y275 of the TPR helix and also to residues K87 and K89, which function as the gatekeepers to select for PlcR/PapR binding only, while the K197 residue interacts with the PapR C-terminus (Declerck *et al*. 2007; Bouillaut *et al*. 2008). The two PapR phenylalanines, which are not always present in PapR peptides in other *Bacillus* species, stabilize the peptide by inserting it into a hydrophobic cleft between α-helices 5 and 7 of PlcR. The processed PapR in all four pentapeptide classes has a proline residue that is predicted to fit the peptide into the binding pocket within the TPR domain of PlcR (Bouillaut *et al*. 2008). After the structure of *B. thuringiensis* PlcR was determined, it was found that the more biologically-relevant PapR sequence is ADLPFEF instead of the shortened pentapeptide LPFEF (Bouillaut *et al*. 2008). The additional two amino acids in the heptapeptide were predicted to fit into the continuation of the groove of the PapR-binding site in PlcRI, and also account for receptor-ligand specificity (Bouillaut *et al*. 2008; Rocha-Estrada *et al*. 2010). Since there is some PapR similarity, cross-talk does exist, but PlcRI in *B. thuringiensis* was shown to be highly activated by its cognate PapR heptapeptide and to a much lesser extent by the other three heptapeptides, while PlcRII and III are more promiscuous (Bouillaut *et al*. 2008). The positioning of the TPR is thought to be influenced by the binding of the ligand, which subsequently affects dimerization and positioning of the DBD to DNA (Declerck *et al*. 2007; Grenha *et al*. 2013). Remarkably, the structures of PlcR in *B. thuringiensis* and PgrX in *Enterococcus faecalis* are extremely similar, even where the peptide signal binds to the receptor, yet these proteins have completely different functions. DNA binding is non-existent in PlcR unless bound to the PapR peptide, while PgrX binds to DNA sans signal (Declerck *et al*. 2007; Bouillaut *et al*. 2008).

### Specificity in two-component Gram-positive peptide QS systems

In addition to cytoplasmic peptide receptors, Gram-positive bacteria also employ membrane-bound receptors for QS communication. These peptide QS receptors usually are histidine kinases that transfer a phosphate to cytoplasmic response regulators (Hoch and Silhavy 1995; Inouye and Dutta 2003; Simon, Crane and Crane 2007), which either initiate or repress transcription of a particular set of genes (Fig. 1D). While the cytoplasmic portions of these membrane-bound receptors belong to the histidine kinase family and share homology, little homology exists in their LBDs, which determine ligand specificity (Magnuson, Solomon and Grossman 1994; Pestova, Havarstein and Morrison 1996; Miller and Bassler 2001; Novick and Geisinger 2008; Ng and Bassler 2009). Since these receptors are membrane-bound, structural analysis is difficult. Here, we will use AgrC as an example to illustrate the mechanisms used by this type of receptor to differentiate between related peptide signals.

*Staphylococcus aureus* is a clinically important human pathogen that uses a two-component accessory gene regulator (Agr) system as the primary QS circuit. Agr controls the expression of several virulence factors that allow this bacterium to persist in almost any human tissue (Novick and Geisinger 2008; Le and Otto 2015). This system is expressed from two divergently transcribed loci: the *agrBDCA* operon (RNAII) and RNAIII (Novick *et al*. 1995; Novick and Geisinger 2008)*.* The *agrC* gene encodes the AgrC transmembrane QS histidine kinase receptor, while *agrA* encodes the cytoplasmic response regulator protein (Lina *et al*. 1998; Peng *et al*. 1988). The *agrD* gene encodes the 46–47 amino acid peptide precursor AgrD, which is further processed by AgrB, a transmembrane endopeptidase, to a 7 to 9 amino acid peptide that contains a conserved thiolactone ring formed between the sulfur atom from a cysteine residue to the C-terminus of the autoinducer peptide (AIP) that is required for its activity (Fig. 1D) (Ji, Beavis and Novick 1995, 1997; Novick *et al*. 1995; Mayville *et al*. 1999; Zhang and Ji 2004; Novick and Geisinger 2008). RNAIII is a non-coding RNA that modulates expression of other regulators (Geisinger *et al*. 2006; Boisset *et al*. 2007; Novick and Geisinger 2008; Gupta, Luong and Lee 2015).

The Agr QS system is conserved among staphylococci, but variations within AgrD and the C-terminus of AgrB result in different mature AIPs. Moreover, polymorphism is observed in the extracellular LBD of AgrC among different Staphylococci strains. Binding of cognate AgrD peptide to AgrC sensor kinase leads to the activation of kinase activity of the receptor, while binding of non-cognate AgrD peptide leads to receptor kinase inhibition (Ji, Beavis and Novick 1997; Mayville *et al*. 1999; Otto *et al*. 2001; Olson *et al*. 2014; Le and Otto 2015).

*Staphylococcus aureus* Agr systems can be classified into four different groups based on variations in the *agrB*, *agrD* and *agrC* genes, with even more variations found in other species as well (Otto *et al*. 1999; Jarraud *et al*. 2000; Lyon and Novick 2004; Novick and Geisinger 2008). All AgrD AIPs have distinctly defined sequences with a conserved cysteine in the fifth position from the C-terminus that serves as the site of the thiolactone bond with the C-terminal carboxyl end of the peptide. AgrD-I is an octapeptide (YSTCDFIM) whose sequence varies from AgrD-IV (YSTCYFIM) with a single residue change; AgrD-II is a nonapeptide with the sequence (GVNACSSLF) and AgrD-III is a heptapeptide (INCDFLL) (Fig. 2D) (Jarraud *et al*. 2000; Lyon *et al*. 2002; Novick and Geisinger 2008). AgrD AIP is found in all known strains of *Staph. intermedius* and contains a serine residue in place of the cysteine residue to form a cyclic lactone instead (Bannoehr *et al*. 2007).

AIP specificity appears to correlate to diseases caused by the different *Staph. aureus* groups in the host. Each of the four *Staph. aureus* groups has a characteristic biological consequence: Group I is linked to enterotoxin disease, Group II is linked to early vancomycin-resistant strains, Groups II and III are linked to endocarditis, Group III is linked to menstrual toxic shock syndrome, and Group IV is linked to exfoliative disease (Jarraud *et al*. 2000, 2002; Sakoulas *et al*. 2002; Novick and Geisinger 2008). Using AgrC chimeras and a battery of AIP molecules, it was determined that the AIP makes two interactions with the AgrC protein. The first is a hydrophobic interaction between AgrC and the C-terminal residues on the AIP. The second interaction is more sequence specific, lending to specificity of the receptor for the AIP. For example, it was shown that the tail region of AgrD-II is required for AgrC-II activation and a mutant peptide with a single residue change (GVAACSSLF) is an inhibitor of all four AgrC classes (Mayville *et al*. 1999). The exocyclic residue N in AgrD-II, and endocyclic residues D and Y in AgrD-I and AgrD-IV, respectively, are critical for cognate AgrC receptor activation. Also, additional residues added to the N-terminal end of AgrD-III abolish receptor activation (Lyon *et al*. 2002). Taken together, these studies have shown that not only that the sequence of the AgrD AIP important for either recognition or inhibition of AgrC, but that stereochemistry can also alter activity.

The AgrC receptor has an N-terminal transmembrane sensor domain plus a C-terminal histidine kinase domain. The C-terminal domain is highly conserved among staphylococci; however, the N-terminal sensor domain is as divergent as *agrD* and *agrB* (Wright *et al*. 2004; Novick and Geisinger 2008). Mutagenesis studies of AgrC concluded that AgrD discrimination likely occurs in the second extracellular loop in the sensor domain between helices three and four. Interestingly, switching five residues that differed in this region between AgrC-I and AgrC-IV switched the specificity of the cognate AgrD AIP (Wright *et al*. 2004). For AgrC-IV, residue T101 is crucial for activation, and making T101A, V107S and I116S mutations are enough to change the specificity of AgrC-IV to that of AgrC-I (Chen, Tsou and Chen 2009). In contrast, mutating AgrC-I residue Y100 to F and I171 to K broadened the specificity of AgrC-I greatly (Geisinger *et al*. 2008; Jensen *et al*. 2008; Novick and Geisinger 2008; Geisinger, Muir and Novick 2009). AgrC-I residue I171 was also deemed a critical inhibitory residue by non-cognate AgrD AIPs (Novick and Geisinger 2008).

### Specificity in two-component Gram-negative QS receptors

While the LuxI-LuxR-type system is common among Gram-negative QS bacteria, some species use membrane-bound receptors for AHSL detection (Fig. 1B). For instance, *V. harveyi* responds to 3OH-C4-HSL, or HAI-1 (Fig. 2A), exclusively using a membrane-bound histidine kinase receptor LuxN (Cao and Meighen 1989; Bassler *et al*. 1993). HAI-1 is made by synthase LuxM, which shares little homology to LuxI (Bassler *et al*. 1993; Bassler, Wright and Silverman 1994; Freeman, Lilley and Bassler 2000; Ng and Bassler 2009). AHSLs with longer acyl tails, or lacking a hydroxyl group at the C3 position of the acyl tail, do not induce QS in *V. harveyi*, suggesting that LuxN is specific for HAI-1 (Ke, Miller and Bassler 2015). Furthermore, HSLs with an eight or longer carbon acyl chain can outcompete HAI-1 as antagonists with increasing potency in accordance with increasing acyl chain length, even without the C3 hydroxyl group (Ke, Miller and Bassler 2015).

It is predicted that LuxN contains nine transmembrane helices, and the HAI-1-binding site lies between helices four and seven (Swem *et al*. 2008). Mutations in LuxN revealed that residue H210 is responsible for recognizing the C3 hydroxyl moiety on the HAI-1 AI but is not important for determining acyl chain length (Ke, Miller and Bassler 2015). Mutations in LuxN residue pair L166H/N176D renders LuxN unable to detect any HAI-1 molecules, while mutations in residue pairs G147D/Y194H, S184I/S230P and T159I/Y193K broadens the specificity of LuxN to detect longer acyl chains up to C10 (Ke, Miller and Bassler 2015). More specifically, LuxN residue L166 discriminates HSLs for ones that have four carbon acyl chains, and decreasing the size of the side chain on residue L166 allowed longer acyl chains to be detected. Taken together, LuxN residues H210 and L166 provide simple, yet rigorous discrimination for the cognate HAI-1, leading to efficient and informative signaling that is not easily interrupted.

Although related to *V. fischeri* and *V. harveyi*, *V. cholerae* does not produce or detect any AHSLs. Instead, this human pathogen possesses a CqsS/CqsA QS system which is conserved in many other *Vibrio* species (Miller *et al*. 2002; Henke and Bassler 2004; Higgins *et al*. 2007; Ng and Bassler 2009). CqsA is the synthase for the signal CAI-1, which is detected by the membrane-bound receptor CqsS. CAI-1 was first purified and identified as (*S*)-3-hydroxytridecan-4-one (Fig. 2C) (Higgins *et al*. 2007). However, CAI-1 is not directly synthesized by CqsA. Instead, CqsA uses decanoyl-CoA and SAM to make the precursor enamino-CAI-1 (Ea-CAI-1), which is further metabolized into CAI-1. Surprisingly, CqsA can also use octanoyl-CoA to make EA-C8-CAI-1; and both Ea-CAI-1and Ea-C8-CAI-1 are potent agonists of CqsS (Fig. 2C) (Higgins *et al*. 2007; Kelly *et al*. 2009; Ng *et al*. 2011; Wei *et al*. 2011).

Since CqsS is predicted to have six transmembrane helices, structural studies are very difficult, if not impossible. Thus, an orthogonal chemical genetic approach was developed to identify key residues important for CAI-1 and CqsS interactions. Specifically, an array of CAI-1 analogs with defined modifications was synthesized and used to screen for CqsS mutants that would respond to these molecules. Using this approach, it was revealed that the highly conserved residues W104 and S107 located in the fourth transmembrane helix serve to distinguish between the hydroxyl and amino moieties at the C3 position on CAI-1 (Ng *et al*. 2010). Two chemically modified CAI-1 analogs were used to probe CqsS specificity for tail length and head group. Phenyl-CAI-1 has a bulky modification on the head group of CAI-1 and functions as a CqsS antagonist, while C8-CAI-1 has a shorter tail length of 8 carbons instead of the 10 carbon tail length seen with CAI-1 and functions as a weak CqsS agonist. By screening for CqsS mutants that could use these analogs but at the same time were insensitive to the native CAI-1 ligand, it was discovered that CqsS residue F162 is important for CAI-1 head group recognition, while residue C170 determines CAI-1 tail length preference (Ng *et al*. 2010). Intriguingly, C170 is only present in the *V. cholerae* CqsS receptor, whereas other CqsS receptors such as the one in *V. harveyi*, have a phenylalanine residue at this position (Ng *et al*. 2010, 2011). This observation infers that other *Vibrio* species recognize CAI-1 molecules with a C8 tail instead of the longer C10 tail, as the phenylalanine in the corresponding position to *V. cholerae* C170 would prevent binding of longer tailed CAI-1 molecules. A more extensive study on ligand specificity revealed that *V. cholerae* mutant CqsS C170F can only detect Ea-C8-CAI-1 and C8-CAI-1, but not CAI-1 or Ea-CAI-1 (Ng *et al*. 2011). As expected, *V. harveyi* CqsS only recognizes Ea-C8-CAI-1 and does not respond to CAI-1 with a C10 tail (Ng *et al*. 2011). Mutating *V. harveyi* CqsS residue F175 (the corresponding position to *V. cholerae* C170) to a cysteine relaxes the specificity of *V. harveyi* CqsS, and the mutant receptor is able to detect CAI-1 and Ea-CAI-1 as well as Ea-C8-CAI-1. Interestingly, *V. harveyi* only makes CAI-1 molecules with C8 tails due to high substrate specificity of its CqsA synthase (Ng *et al*. 2011). Thus, signal production and signal detection in the CqsA/CqsS system in these two *Vibrio* species have co-evolved. While it is likely that both receptors emerged from a common ancestor, the divergence in specificity of these two *Vibrio* QS systems could be due to selective advantages that require relaxed specificity in *V. cholerae* and more stringent requirements in *V. harveyi*.

## COMPLEXITY IN QS NETWORK ARCHITECTURE

**Figure 3.**
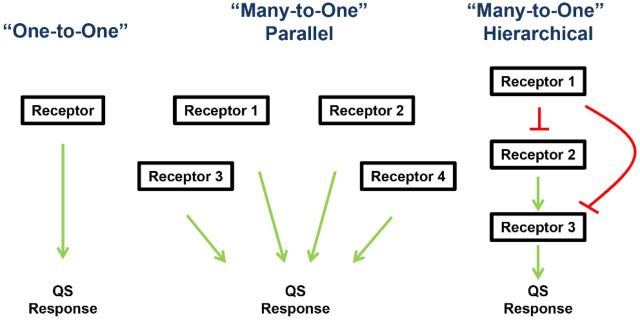
Different QS network configurations. In a ‘One-to-One’ system, a single receptor controls the entire QS response. In a ‘Many-to-One’ parallel circuit, information contained in multiple autoinducers are integrated together to control the QS response. In a ‘Many-to-One’ hierarchical system, many QS receptors are connected in a signaling cascade in which the downstream receptor activity is controlled by the upstream receptors. Arrows and T-bar denote hypothetical activation and repression pattern, respectively.

As illustrated above, intrinsic receptor ligand specificity plays an important role in determining the final QS output in response to cognate and non-cognate signals. This is especially important when certain QS bacteria employ a ‘one-to-one’ QS network configuration, in which the overall QS response is solely controlled by a single receptor responding to a single signal (Fig. 3). Yet, it is not uncommon for different QS signal transduction pathways to be inter-connected into a complex QS network. A few of these bacterial QS systems that have a parallel (or ‘many-to-one’) or hierarchical configuration will be examined below (Fig. 3). We will discuss how varying architectures are advantageous for achieving different biological goals.

### Parallel QS systems in *Vibrio* species

Parallel network architectures that utilize a phospho-relay signal transduction mechanism are common in *Vibrio* QS systems. While *V. fischeri* contains a LuxI-LuxR-type QS system, *V. cholerae* and *V. harveyi* do not utilize this type of receptor for AI detection. QS networks in these two species are composed of multiple histidine kinase receptors converging to control a single regulator that governs the downstream QS response (Fig. 1B). Although network architecture is similar between the two *Vibrio* species, the exact identities of the receptors used differ (Miller *et al*. 2002; Zhu *et al*. 2002; Henke and Bassler 2004; Lenz *et al*. 2004; Long *et al*. 2009). In *V. harveyi*, three QS receptors LuxN, LuxPQ and CqsS, which are all transmembrane histidine kinases, convey their AI information to LuxO through LuxU (Bassler, Wright and Silverman 1994; Freeman, Lilley and Bassler 2000; Henke and Bassler 2004; Neiditch *et al*. 2005, 2006; Higgins *et al*. 2007; Swem *et al*. 2008). In contrast, *V. cholerae* possesses four QS receptors, three of which are transmembrane histidine kinases, LuxPQ, CqsS and CqsR, while the fourth is predicted to be a cytoplasmic receptor VpsS (Miller and Bassler 2001; Miller *et al*. 2002; Waters and Bassler 2005; Ng and Bassler 2009; Jung, Chapman and Ng 2015). It should be noted that, in addition to the LuxI-LuxR system, *V. fischeri* also carries membrane-bound LuxPQ and AinN receptors for QS gene regulation (Kuo, Callahan and Dunlap 1996; Schaefer *et al*. 1996a; Lupp *et al*. 2003). Kinase activity of all of these receptors predominates when AI concentration is low; LuxO is activated by phosphorylation, resulting in a low cell density gene expression program. When AI concentration is high, receptor phosphatase activity predominates and LuxO becomes inactive, resulting in a high cell density gene expression program.

### Distinct information contained within each signal in *Vibrio* QS Systems

Although it seems perplexing that LuxO is controlled by multiple redundant receptors, it is possible that each signal could be translated into a particular output response. In other words, each detected signal may have its own meaning (Bassler, Wright and Silverman 1994; Miller *et al*. 2002; Henke and Bassler 2004; Ng *et al*. 2011). For example, *V*. *harveyi* detects three AIs HAI-1, CAI-1 and AI-2, using receptors LuxN, CqsS and LuxPQ, respectively. These systems are thought to be used for intra-species, intra-genus, and inter-species communication (Federle and Bassler 2003; Henke and Bassler 2004; Ng *et al*. 2011). HAI-1 is exclusively made and detected by *V. harveyi*. CAI-1 and its related molecules are made and detected by many *Vibrio* species. As discussed above, CAI-1 with an 8- or 10-carbon tail length regulates *V. cholerae* CqsS kinase activity, while CAI-1 with an 8-carbon tail length controls *V. harveyi* CqsS (Ng *et al*. 2011). Thus, CAI-1 with an 8-carbon tail length may be used for inter-*Vibrio* signaling, while CAI-1 with a 10-carbon tail length could be a *V. cholerae*-specific QS signal. AI-2 is made by a variety of bacterial species carrying the gene *luxS* and therefore LuxPQ could be used for *Vibrio*s to enumerate the abundance of surrounding microbial species (Bassler, Wright and Silverman 1994; Surette, Miller and Bassler 1999; Chen *et al*. 2002; Miller *et al*. 2002; Ng *et al*. 2011; Pereira, Thompson and Xavier 2013). Although the signals that regulate the recently-identified receptors CqsR and VpsS are unknown, it is likely that these two receptors also detect small chemical molecules that are unique from one another (Jung, Chapman and Ng 2015). As a result, specific information contained in each signal may allow for subtle behavioral changes that are more suitable for adapting to certain environmental niches.

### Parallel inputs prevent premature QS induction in *Vibrio cholerae*

In *V. cholerae*, the loss of three of the four QS receptor histidine kinase activities has little effect on *V*. *cholerae* colonization of animal hosts. Thus, any one of its four QS receptors acting alone is sufficient to regulate gene expression in response to cell density (Jung, Chapman and Ng 2015). In contrast, mutants lacking all four QS receptors do not colonize the infant mouse host. Even though having four receptors seems redundant, *V. cholerae* inhabits vastly different environmental niches and it may encounter a variety of different bacterial species that produce their own array of signaling molecules. In contrast to *V. harveyi*, where QS can be induced by addition of a single AI such as HAI-1 (Bassler *et al*. 1993; Bassler, Wright and Silverman 1994; Mok, Wingreen and Bassler 2003; Henke and Bassler 2004), it was shown that excess CAI-1 added to *V. cholerae* does not induce QS prematurely (Jung, Chapman and Ng 2015). Based on these findings, it is thought that these receptors may act together to prevent a premature committed responses caused by signal perturbations or, possibly, analogous molecules associated with environmental changes and noise (Fig. 3) (Ng *et al*. 2011, 2012; Jung, Chapman and Ng 2015). Previous studies have shown that molecules with structures drastically different from CAI-1 could control CqsS activity (Ng *et al*. 2012), suggesting that there are possibilities for decoy molecules acting alone on a single QS receptor. It should be noted that CqsS of *V. harveyi* has higher ligand detection specificity than that of *V. cholerae* and is less likely to be affected by signal perturbation (Ng *et al*. 2011). Therefore, even though these *Vibrio* species utilize parallel signaling networks, their biological goals achieved are different. The *V. harveyi* QS system is proposed to function as a ‘coincidence detector’ (Mok, Wingreen and Bassler 2003; Henke and Bassler 2004), while the *V. cholerae* redundant receptors function together in parallel to resist signal perturbations. Taken together, this particular circuit architecture could be crucial in maintaining population-wide expression of QS genes, and it could also function in preventing premature commitment to high cell density gene expression until the cells are ready and when the response is appropriate.

### Hierarchical QS systems in *Pseudomonas aeruginosa*

In addition to parallel signal transduction pathways, different network configurations have been identified in other QS systems (Fig. 3). For instance, the *P. aeruginosa* QS network adopts a unique, hierarchical architecture that consists of two intimately-connected pathways: the LasI-LasR system which makes and detects 3-oxo-C12-HSL, and the RhlI-RhlR system which makes and detects C4-HSL (Whitehead *et al*. 2001; Fuqua and Greenberg 2002). Together, these two QS systems regulate expression of >300 genes involved in virulence factor production and biofilm formation in this pathogen (Schuster *et al*. 2003; Schuster, Urbanowski and Greenberg 2004; Wagner, Gillis and Iglewski 2004; Wei and Ma 2013). While each of these two QS systems regulates a specific set of genes, there exists some overlap. This is because the LasI-LasR circuit is hierarchically positioned to regulate expression of the RhlI-RhlR circuit. LasR, in the presence of 3-oxo-C12-HSL, activates expression of *rhlI and rhlR* (Latifi *et al*. 1996; Pesci *et al*. 1997; Medina *et al*. 2003a,b; Gilbert *et al*. 2009). This network arrangement allows temporal expression of different sets of genes in the *P. aeruginosa* QS regulon (Schuster *et al*. 2003; Smith and Iglewski 2003; Schuster, Urbanowski and Greenberg 2004; Venturi 2006). Additionally, the orphan QscR regulator functions as a negative regulator to repress LasI and RhlI expression (Chugani *et al*. 2001). QscR responds to 3-oxo-C12-HSL and a variety of AHSLs. Therefore, through QscR, the QS response of *P. aeruginosa* could be modulated by its own AHSL feedback or by the presence of other AHSL-producing species (Williams *et al*. 2000; Chugani *et al*. 2001; Ledgham *et al*. 2003; Parsek and Greenberg 2005; Waters and Bassler 2005; Lee, Lequette and Greenberg 2006; Lequette *et al*. 2006; Mattmann and Blackwell 2010).

In addition to network hierarchy, the two *P. aeruginosa* AHSL ligands have different decaying rates, and *P. aeruginosa* behaves differently with different combinatorial (non-additive) concentrations of the two AIs (Cornforth *et al*. 2014). These researchers suggest that signal concentration threshold gates correspond to different responses, such that bacteria can infer both social (cell density) and physical (mass-transfer) environments. Accordingly, by utilizing multiple signals and different combinatorial signal concentrations, personal environments are more fully resolved and result in more complex gene expression patterns within individual cells (Cornforth *et al*. 2014; Drees *et al*. 2014). Finally, there has been an increasing evidence that nutritional cues within infection environments can alter this complex hierarchy (Welsh and Blackwell 2016).

### Competition between two related QS regulatory Rgg systems

Signal antagonism is also incorporated into complex QS systems to dampen output responses. For instance, *Streptococcus pyogenes* regulates its QS response through four different Rgg regulators. Each Rgg receptor presumably responds to a specific peptide signal called SHP (short hydrophobic peptide) and XIP (SigX inducing peptide) (Ibrahim *et al*. 2007a,b; Fontaine *et al*. 2010; Mashburn-Warren, Morrison and Federle 2010, 2012; Chang *et al*. 2011; Fleuchot *et al*. 2011). Of particular interest is the antagonistic action discovered between the Rgg2 and Rgg3 proteins, which respectively respond to SHP2 and SHP3 peptide pheromones (Chang *et al*. 2011; LaSarre, Chang and Federle 2013). While Rgg2 is a transcriptional activator, Rgg3 represses the same target genes. Both regulators bind to a conserved DNA motif present in the promoters of these target genes, including the genes encoding SHP2 and SHP3. Since they share binding sites, only one Rgg protein may bind to the same promoter. Additionally, SHP concentration can skew binding to favor Rgg2. Rgg2 binding induces QS, while Rgg3 binding results in QS repression by monopolizing binding sites essential for SHP production (Lasarre, Aggarwal and Federle 2013; Aggarwal *et al*. 2014). Taken together, Rgg3 binding antagonizes Rgg2 binding and, therefore, may protect against premature induction of the QS circuit in *S. pyogenes* (Aggarwal *et al*. 2014).

### Newly acquired receptors and facultative cheating

A recent study provided insight on the selective advantage of utilizing multiple receptors for the QS response (Travisano and Velicer 2004; Even-Tov *et al*. 2016). The central hypothesis of this work is that strains that have acquired new QS receptors are selected via facultative cheating. Cheaters are individuals that do not contribute to the group but reap the benefits of the cooperative members of the group. From the perspective of QS, cheaters are strains that do not respond to the AI signals due to various mechanisms such as missing a functional QS system. When present in low number within a population, cheaters could exhibit higher fitness under certain growth conditions (Travisano and Velicer 2004; West *et al*. 2006; Popat *et al*. 2012). Moreover, cheating can be either obligate or facultative. In the latter case, facultative cheaters will cooperate with their own kind but cheat when they are in a group of genetically dissimilar individuals (Travisano and Velicer 2004). It is predicted that evolved strains with multiple QS systems are less cooperative than their ancestral strains that have fewer QS receptors because the concentration of the cognate signal of the new receptor is relatively low. However, these new, evolved strains will cooperate in a homogenous population with cells bearing the same number of receptors (Travisano and Velicer 2004; Even-Tov *et al*. 2016). Facultative cheating in these strains with higher number of QS receptors enable these individuals to invest less (e.g. producing less public goods) but maintain the same level of fitness in the presence of its ancestor and yet remain cooperative when the population becomes clonal. Based on this model, in a parallel QS circuit, the newly-introduced receptor must be able to repress its QS response in the absence of cognate signal (such as the QS receptors in the *Vibrio* QS system), while receptors that positively regulate QS could potentially be favored in a hierarchical network (Even-Tov *et al*. 2016).

### Population heterogeneity

The phrase ‘phenotypic heterogeneity’ describes phenotypic variations observed between individual bacterial cells in what was thought to be a genetically homogenous population (Davidson and Surette 2008). In *V. harveyi*, the presence of multiple QS systems renders the population more homogenous in a laboratory batch culture (Plener *et al*. 2015). Potentially, different combinations of AI abundances could lead to heterogeneity of the QS response in *V. harveyi*. Although QS is thought to promote unison behavior, diversification in QS output responses could be beneficial and necessary for the group's survival and well-being (Anetzberger, Schell and Jung 2012). Variation of gene expression profiles in a given population likely depends on the micro-environments encountered by different cells in the community such as stratification within a biofilm (Vlamakis *et al*. 2008; Bischofs *et al*. 2009). For example, *Bacillus subtilis* cells that are motile, biofilm-producing and sporulating localize to specific niches within a biofilm structure, and this localization and ratio of each cell type is temporally and spatially dynamic within the population (Vlamakis *et al*. 2008). Cell-to-cell heterogeneity has also been reported in *Listeria monocytogenes* biofilm communities (Garmyn *et al*. 2011) and in deep tissue infected with *Yersinia pseudotuberculosis* (Davis, Mohammadi and Isberg 2015). Specialized differentiated populational behaviors may allow for cells to react better to changes in their environmental surroundings (Bischofs *et al*. 2009). Therefore, these multiple signal transduction systems are crucial to integrate environmental signals based on spatiotemporal community structure, which differs among neighboring subpopulations, as is seen in biofilm structures (Bischofs *et al*. 2009). Within these biofilm communities, varying AI concentrations could represent different stages of biofilm development, whereby multiple systems are used for a multi-developmental program, where different genes are regulated at differential expression levels (Mehta *et al*. 2009).

## CONCLUSIONS

It is fascinating to consider the evolution of the vastly different bacterial QS systems. Even in related species with similar QS network architectures, the functional goals of these signaling pathways could be vastly different. On the other hand, recurring patterns and characteristics including ligand specificity, evolution and the inter-connection of multiple pathways are observed in unrelated systems. All in all, bacteria have adopted various elegant mechanisms to resist signal antagonism and perturbations. In the last few decades, we have uncovered a vast knowledge about the components and basic concepts in building a QS system; yet, there are many more questions that remain: what governs the transition from a simple ancestral QS system to a complex QS network that we observe in modern bacterial species today? Do different environmental niches determine QS ligand specificity or QS network architecture? How do micro-environments influence QS behavior in a bacterial community? Will and can QS networks become a focus for therapeutic development? We hope that continued research on these topics will provide answers to these important questions.

## References

[bib1] Aggarwal C, Jimenez JC, Nanavati D (2014). Multiple length peptide-pheromone variants produced by *Streptococcus pyogenes* directly bind Rgg proteins to confer transcriptional regulation. J Biol Chem.

[bib2] Anetzberger C, Schell U, Jung K (2012). Single cell analysis of *Vibrio harveyi* uncovers functional heterogeneity in response to quorum sensing signals. BMC Microbiology.

[bib3] Aravind L, Anantharaman V, Balaji S (2005). The many faces of the helix-turn-helix domain: transcription regulation and beyond. FEMS Microbiol Rev.

[bib4] August PR, Grossman TH, Minor C (2000). Sequence analysis and functional characterization of the violacein biosynthetic pathway from *Chromobacterium violaceum*. J Mol Microb Biotech.

[bib5] Bae T, Clerc-Bardin S, Dunny GM (2000). Analysis of expression of *prgX*, a key negative regulator of the transfer of the *Enterococcus faecalis* pheromone-inducible plasmid pCF10. J Mol Biol.

[bib6] Bannoehr J, Ben Zakour NL, Waller AS (2007). Population genetic structure of the *Staphylococcus intermedius* group: insights into agr diversification and the emergence of methicillin-resistant strains. J Bacteriol.

[bib7] Bassler B, Vogel J (2013). Bacterial regulatory mechanisms: the gene and beyond. Curr Opin Microbiol.

[bib8] Bassler BL, Wright M, Showalter RE (1993). Intercellular signalling in *Vibrio harveyi*: sequence and function of genes regulating expression of luminescence. Mol Microbiol.

[bib9] Bassler BL, Wright M, Silverman MR (1994). Multiple signalling systems controlling expression of luminescence in *Vibrio harveyi*: sequence and function of genes encoding a second sensory pathway. Mol Microbiol.

[bib10] Bischofs IB, Hug JA, Liu AW (2009). Complexity in bacterial cell-cell communication: quorum signal integration and subpopulation signaling in the *Bacillus subtilis* phosphorelay. P Natl Acad Sci USA.

[bib11] Blatch GL, Lassle M (1999). The tetratricopeptide repeat: a structural motif mediating protein-protein interactions. Bioessays.

[bib12] Boettcher KJ, Ruby EG (1995). Detection and quantification of *Vibrio fischeri* autoinducer from symbiotic squid light organs. J Bacteriol.

[bib13] Boisset S, Geissmann T, Huntzinger E (2007). *Staphylococcus aureus* RNAIII coordinately represses the synthesis of virulence factors and the transcription regulator Rot by an antisense mechanism. Gene Dev.

[bib14] Bottomley MJ, Muraglia E, Bazzo R (2007). Molecular insights into quorum sensing in the human pathogen *Pseudomonas aeruginosa* from the structure of the virulence regulator LasR bound to its autoinducer. J Biol Chem.

[bib15] Bouillaut L, Perchat S, Arold S (2008). Molecular basis for group-specific activation of the virulence regulator PlcR by PapR heptapeptides. Nucleic Acids Res.

[bib16] Brachmann AO, Brameyer S, Kresovic D (2013). Pyrones as bacterial signaling molecules. Nat Chem Biol.

[bib17] Brameyer S, Heermann R (2015). Specificity of Signal-Binding via Non-AHL LuxR-Type Receptors. PLoS One.

[bib18] Brint JM, Ohman DE (1995). Synthesis of multiple exoproducts in *Pseudomonas aeruginosa* is under the control of RhlR-RhlI, another set of regulators in strain PAO1 with homology to the autoinducer-responsive LuxR-LuxI family. J Bacteriol.

[bib19] Cao JG, Meighen EA (1989). Purification and structural identification of an autoinducer for the luminescence system of *Vibrio harveyi*. J Biol Chem.

[bib20] Chang JC, LaSarre B, Jimenez JC (2011). Two group A streptococcal peptide pheromones act through opposing Rgg regulators to control biofilm development. PLoS Pathog.

[bib21] Chen G, Swem LR, Swem DL (2011). A strategy for antagonizing quorum sensing. Mol Cell.

[bib22] Chen LC, Tsou LT, Chen FJ (2009). Ligand-receptor recognition for activation of quorum sensing in *Staphylococcus aureus*. J Microbiol.

[bib23] Chen X, Schauder S, Potier N (2002). Structural identification of a bacterial quorum-sensing signal containing boron. Nature.

[bib24] Choi SH, Greenberg EP (1991). The C-terminal region of the *Vibrio fischeri* LuxR protein contains an inducer-independent *lux* gene activating domain. P Natl Acad Sci USA.

[bib25] Choi SH, Greenberg EP (1992). Genetic dissection of DNA binding and luminescence gene activation by the *Vibrio fischeri* LuxR protein. J Bacteriol.

[bib26] Christie PJ. (1997). *Agrobacterium tumefaciens* T-complex transport apparatus: a paradigm for a new family of multifunctional transporters in eubacteria. J Bacteriol.

[bib27] Chugani SA, Whiteley M, Lee KM (2001). QscR, a modulator of quorum-sensing signal synthesis and virulence in *Pseudomonas aeruginosa*. P Natl Acad Sci USA.

[bib28] Collins CH, Arnold FH, Leadbetter JR (2005). Directed evolution of *Vibrio fischeri* LuxR for increased sensitivity to a broad spectrum of acyl-homoserine lactones. Mol Microbiol.

[bib29] Collins CH, Leadbetter JR, Arnold FH (2006). Dual selection enhances the signaling specificity of a variant of the quorum-sensing transcriptional activator LuxR. Nat Biotechnol.

[bib30] Cook LC, Federle MJ (2014). Peptide pheromone signaling in *Streptococcus* and *Enterococcus*. FEMS Microbiol Rev.

[bib31] Cornforth DM, Popat R, McNally L (2014). Combinatorial quorum sensing allows bacteria to resolve their social and physical environment. P Natl Acad Sci USA.

[bib32] Davidson CJ, Surette MG (2008). Individuality in bacteria. Annu Rev Genet.

[bib33] Davis KM, Mohammadi S, Isberg RR (2015). Community behavior and spatial regulation within a bacterial microcolony in deep tissue sites serves to protect against host attack. Cell Host Microbe.

[bib34] Declerck N, Bouillaut L, Chaix D (2007). Structure of PlcR: insights into virulence regulation and evolution of quorum sensing in Gram-positive bacteria. P Natl Acad Sci USA.

[bib35] Drees B, Reiger M, Jung K (2014). A modular view of the diversity of cell-density-encoding schemes in bacterial quorum-sensing systems. Biophys J.

[bib36] Dufour D, Levesque CM (2013). Bacterial behaviors associated with the quorum-sensing peptide pheromone ('alarmone') in streptococci. Future Microbiol.

[bib37] Eberhard A, Burlingame AL, Eberhard C (1981). Structural identification of autoinducer of *Photobacterium fischeri* luciferase. Biochemistry.

[bib38] Engebrecht J, Nealson K, Silverman M (1983). Bacterial bioluminescence: isolation and genetic analysis of functions from *Vibrio fischeri*. Cell.

[bib39] Engebrecht J, Silverman M (1984). Identification of genes and gene products necessary for bacterial bioluminescence. P Natl Acad Sci USA.

[bib40] Even-Tov E, Omer Bendori S, Valastyan J (2016). Social evolution selects for redundancy in bacterial quorum sensing. PLoS Biol.

[bib41] Federle MJ, Bassler BL (2003). Interspecies communication in bacteria. Journal of clinical investigation.

[bib42] Fleuchot B, Gitton C, Guillot A (2011). Rgg proteins associated with internalized small hydrophobic peptides: a new quorum-sensing mechanism in streptococci. Mol Microbiol.

[bib43] Fontaine L, Boutry C, de Frahan MH (2010). A novel pheromone quorum-sensing system controls the development of natural competence in *Streptococcus thermophilus* and *Streptococcus salivarius*. J Bacteriol.

[bib44] Freeman JA, Lilley BN, Bassler BL (2000). A genetic analysis of the functions of LuxN: a two-component hybrid sensor kinase that regulates quorum sensing in *Vibrio harveyi*. Mol Microbiol.

[bib45] Fuqua C, Greenberg EP (1998). Self perception in bacteria: quorum sensing with acylated homoserine lactones. Curr Opin Microbiol.

[bib46] Fuqua C, Greenberg EP (2002). Listening in on bacteria: acyl-homoserine lactone signalling. Nat Rev Mol Cell Bio.

[bib47] Fuqua WC, Winans SC (1994). A LuxR-LuxI type regulatory system activates *Agrobacterium* Ti plasmid conjugal transfer in the presence of a plant tumor metabolite. J Bacteriol.

[bib48] Garmyn D, Gal L, Briandet R (2011). Evidence of autoinduction heterogeneity via expression of the Agr system of *Listeria monocytogenes* at the single-cell level. Appl Environ Microb.

[bib49] Geisinger E, Adhikari RP, Jin R (2006). Inhibition of rot translation by RNAIII, a key feature of agr function. Mol Microbiol.

[bib50] Geisinger E, George EA, Chen J (2008). Identification of ligand specificity determinants in AgrC, the *Staphylococcus aureus* quorum-sensing receptor. J Biol Chem.

[bib51] Geisinger E, Muir TW, Novick RP (2009). Agr receptor mutants reveal distinct modes of inhibition by Staphylococcal autoinducing peptides. P Natl Acad Sci USA.

[bib52] Gilbert KB, Kim TH, Gupta R (2009). Global position analysis of the *Pseudomonas aeruginosa* quorum-sensing transcription factor LasR. Mol Microbiol.

[bib53] Gohar M, Faegri K, Perchat S (2008). The PlcR virulence regulon of *Bacillus cereus*. PLoS One.

[bib54] Gominet M, Slamti L, Gilois N (2001). Oligopeptide permease is required for expression of the *Bacillus thuringiensis* plcR regulon and for virulence. Mol Microbiol.

[bib55] Grenha R, Slamti L, Nicaise M (2013). Structural basis for the activation mechanism of the PlcR virulence regulator by the quorum-sensing signal peptide PapR. P Natl Acad Sci USA.

[bib56] Gupta RK, Luong TT, Lee CY (2015). RNAIII of the *Staphylococcus aureus* agr system activates global regulator MgrA by stabilizing mRNA. P Natl Acad Sci USA.

[bib57] Hanzelka BL, Greenberg EP (1995). Evidence that the N-terminal region of the *Vibrio fischeri* LuxR protein constitutes an autoinducer-binding domain. J Bacteriol.

[bib58] Hanzelka BL, Parsek MR, Val DL (1999). Acylhomoserine lactone synthase activity of the *Vibrio fischeri* AinS protein. J Bacteriol.

[bib59] Havarstein LS, Coomaraswamy G, Morrison DA (1995). An unmodified heptadecapeptide pheromone induces competence for genetic transformation in *Streptococcus pneumoniae*. P Natl Acad Sci USA.

[bib60] Hawkins AC, Arnold FH, Stuermer R (2007). Directed evolution of *Vibrio fischeri* LuxR for improved response to butanoyl-homoserine lactone. Appl Environ Microb.

[bib61] Henke JM, Bassler BL (2004). Three parallel quorum-sensing systems regulate gene expression in *Vibrio harveyi*. J Bacteriol.

[bib62] Higgins DA, Pomianek ME, Kraml CM (2007). The major *Vibrio cholerae* autoinducer and its role in virulence factor production. Nature.

[bib63] Hoch J, Silhavy T (1995). Two-Component Signal Transduction.

[bib64] Hwang I, Li PL, Zhang L (1994). TraI, a LuxI homologue, is responsible for production of conjugation factor, the Ti plasmid N-acylhomoserine lactone autoinducer. P Natl Acad Sci USA.

[bib65] Ibrahim M, Guillot A, Wessner F (2007a). Control of the transcription of a short gene encoding a cyclic peptide in *Streptococcus thermophilus*: a new quorum-sensing system?. J Bacteriol.

[bib66] Ibrahim M, Nicolas P, Bessieres P (2007b). A genome-wide survey of short coding sequences in streptococci. Microbiology.

[bib67] Inouye M, Dutta R (2003). Histidine Kinases in Signal Transduction.

[bib68] Jarraud S, Lyon GJ, Figueiredo AM (2000). Exfoliatin-producing strains define a fourth agr specificity group in *Staphylococcus aureus*. J Bacteriol.

[bib69] Jarraud S, Mougel C, Thioulouse J (2002). Relationships between *Staphylococcus aureus* genetic background, virulence factors, *agr* groups (alleles), and human disease. Infect Immun.

[bib70] Jensen RO, Winzer K, Clarke SR (2008). Differential recognition of *Staphylococcus aureus* quorum-sensing signals depends on both extracellular loops 1 and 2 of the transmembrane sensor AgrC. J Mol Biol.

[bib71] Ji G, Beavis R, Novick RP (1997). Bacterial interference caused by autoinducing peptide variants. Science.

[bib72] Ji G, Beavis RC, Novick RP (1995). Cell density control of staphylococcal virulence mediated by an octapeptide pheromone. P Natl Acad Sci USA.

[bib73] Jimenez JC, Federle MJ (2014). Quorum sensing in group A *Streptococcus*. Front Cell Infect Microbiol.

[bib74] Jung SA, Chapman CA, Ng WL (2015). Quadruple quorum-sensing inputs control *Vibrio cholerae* virulence and maintain system robustness. PLoS Pathog.

[bib75] Ke X, Miller LC, Bassler BL (2015). Determinants governing ligand specificity of the *Vibrio harveyi* LuxN quorum-sensing receptor. Mol Microbiol.

[bib76] Kelly RC, Bolitho ME, Higgins DA (2009). The *Vibrio cholerae* quorum-sensing autoinducer CAI-1: analysis of the biosynthetic enzyme CqsA. Nat Chem Biol.

[bib77] Kleerebezem M, Quadri LE, Kuipers OP (1997). Quorum sensing by peptide pheromones and two-component signal-transduction systems in Gram-positive bacteria. Mol Microbiol.

[bib78] Kuo A, Callahan SM, Dunlap PV (1996). Modulation of luminescence operon expression by N-octanoyl-L-homoserine lactone in *ainS* mutants of *Vibrio fischeri*. J Bacteriol.

[bib79] Lasarre B, Aggarwal C, Federle MJ (2013). Antagonistic Rgg regulators mediate quorum sensing via competitive DNA binding in *Streptococcus pyogenes*. MBio.

[bib80] LaSarre B, Chang JC, Federle MJ (2013). Redundant group a *streptococcus* signaling peptides exhibit unique activation potentials. J Bacteriol.

[bib81] Latifi A, Foglino M, Tanaka K (1996). A hierarchical quorum-sensing cascade in *Pseudomonas aeruginosa* links the transcriptional activators LasR and RhIR (VsmR) to expression of the stationary-phase sigma factor RpoS. Mol Microbiol.

[bib82] Le KY, Otto M (2015). Quorum-sensing regulation in staphylococci-an overview. Front Microbiol.

[bib83] Ledgham F, Ventre I, Soscia C (2003). Interactions of the quorum sensing regulator QscR: interaction with itself and the other regulators of *Pseudomonas aeruginosa* LasR and RhlR. Mol Microbiol.

[bib84] Lee JH, Lequette Y, Greenberg EP (2006). Activity of purified QscR, a *Pseudomonas aeruginosa* orphan quorum-sensing transcription factor. Mol Microbiol.

[bib85] Lenz DH, Mok KC, Lilley BN (2004). The small RNA chaperone Hfq and multiple small RNAs control quorum sensing in *Vibrio harveyi* and *Vibrio cholerae*. Cell.

[bib86] Lequette Y, Lee JH, Ledgham F (2006). A distinct QscR regulon in the *Pseudomonas aeruginosa* quorum-sensing circuit. J Bacteriol.

[bib87] Lereclus D, Agaisse H, Gominet M (1996). Identification of a *Bacillus thuringiensis* gene that positively regulates transcription of the phosphatidylinositol-specific phospholipase C gene at the onset of the stationary phase. J Bacteriol.

[bib88] Lina G, Jarraud S, Ji G (1998). Transmembrane topology and histidine protein kinase activity of AgrC, the agr signal receptor in *Staphylococcus aureus*. Mol Microbiol.

[bib89] Long T, Tu KC, Wang Y (2009). Quantifying the integration of quorum-sensing signals with single-cell resolution. PLoS Biol.

[bib90] Lupp C, Urbanowski M, Greenberg EP (2003). The *Vibrio fischeri* quorum-sensing systems *ain* and *lux* sequentially induce luminescence gene expression and are important for persistence in the squid host. Mol Microbiol.

[bib91] Lyon GJ, Novick RP (2004). Peptide signaling in *Staphylococcus aureus* and other Gram-positive bacteria. Peptides.

[bib92] Lyon GJ, Wright JS, Muir TW (2002). Key determinants of receptor activation in the *agr* autoinducing peptides of *Staphylococcus aureus*. Biochemistry.

[bib93] McClean KH, Winson MK, Fish L (1997). Quorum sensing and *Chromobacterium violaceum*: exploitation of violacein production and inhibition for the detection of *N*-acylhomoserine lactones. Microbiology.

[bib94] Magnuson R, Solomon J, Grossman AD (1994). Biochemical and genetic characterization of a competence pheromone from *B. subtilis*. Cell.

[bib95] Mashburn-Warren L, Morrison DA, Federle MJ (2010). A novel double-tryptophan peptide pheromone controls competence in *Streptococcus* spp. via an Rgg regulator. Mol Microbiol.

[bib96] Mashburn-Warren L, Morrison DA, Federle MJ (2012). The cryptic competence pathway in *Streptococcus pyogenes* is controlled by a peptide pheromone. J Bacteriol.

[bib97] Mattmann ME, Blackwell HE (2010). Small molecules that modulate quorum sensing and control virulence in *Pseudomonas aeruginosa*. J Org Chem.

[bib98] Mayville P, Ji G, Beavis R (1999). Structure-activity analysis of synthetic autoinducing thiolactone peptides from *Staphylococcus aureus* responsible for virulence. P Natl Acad Sci USA.

[bib99] Medina G, Juarez K, Diaz R (2003a). Transcriptional regulation of *Pseudomonas aeruginosa rhlR*, encoding a quorum-sensing regulatory protein. Microbiology.

[bib100] Medina G, Juarez K, Valderrama B (2003b). Mechanism of *Pseudomonas aeruginosa* RhlR transcriptional regulation of the *rhlAB* promoter. J Bacteriol.

[bib101] Mehta P, Goyal S, Long T (2009). Information processing and signal integration in bacterial quorum sensing. Mol Syst Biol.

[bib102] Miller MB, Bassler BL (2001). Quorum sensing in bacteria. Annu Rev Microbiol.

[bib103] Miller MB, Skorupski K, Lenz DH (2002). Parallel quorum sensing systems converge to regulate virulence in *Vibrio cholerae*. Cell.

[bib104] Mok KC, Wingreen NS, Bassler BL (2003). *Vibrio harveyi* quorum sensing: a coincidence detector for two autoinducers controls gene expression. EMBO J.

[bib105] Monnet V, Juillard V, Gardan R (2014). Peptide conversations in Gram-positive bacteria. Crit Rev Microbiol.

[bib106] Nealson KH, Hastings JW (1979). Bacterial bioluminescence: its control and ecological significance. Microbiol Rev.

[bib107] Nealson KH, Platt T, Hastings JW (1970). Cellular control of the synthesis and activity of the bacterial luminescent system. J Bacteriol.

[bib108] Neiditch MB, Federle MJ, Miller ST (2005). Regulation of LuxPQ receptor activity by the quorum-sensing signal autoinducer-2. Mol Cell.

[bib109] Neiditch MB, Federle MJ, Pompeani AJ (2006). Ligand-induced asymmetry in histidine sensor kinase complex regulates quorum sensing. Cell.

[bib110] Ng WL, Bassler BL (2009). Bacterial quorum-sensing network architectures. Annu Rev Genet.

[bib111] Ng WL, Perez L, Cong J (2012). Broad spectrum pro-quorum-sensing molecules as inhibitors of virulence in vibrios. PLoS Pathog.

[bib112] Ng WL, Perez LJ, Wei Y (2011). Signal production and detection specificity in *Vibrio* CqsA/CqsS quorum-sensing systems. Mol Microbiol.

[bib113] Ng WL, Wei Y, Perez LJ (2010). Probing bacterial transmembrane histidine kinase receptor-ligand interactions with natural and synthetic molecules. P Natl Acad Sci USA.

[bib114] Nguyen Y, Nguyen NX, Rogers JL (2015). Structural and mechanistic roles of novel chemical ligands on the SdiA quorum-sensing transcription regulator. MBio.

[bib115] Novick RP, Geisinger E (2008). Quorum sensing in staphylococci. Annu Rev Genet.

[bib116] Novick RP, Projan SJ, Kornblum J (1995). The *agr* P2 operon: an autocatalytic sensory transduction system in *Staphylococcus aureus*. Mol Gen Genet.

[bib117] Ochsner UA, Koch AK, Fiechter A (1994). Isolation and characterization of a regulatory gene affecting rhamnolipid biosurfactant synthesis in *Pseudomonas aeruginosa*. J Bacteriol.

[bib118] Ochsner UA, Reiser J (1995). Autoinducer-mediated regulation of rhamnolipid biosurfactant synthesis in *Pseudomonas aeruginosa*. P Natl Acad Sci USA.

[bib119] Olson ME, Todd DA, Schaeffer CR (2014). *Staphylococcus epidermidis agr* quorum-sensing system: signal identification, cross talk, and importance in colonization. J Bacteriol.

[bib120] Otto M, Echner H, Voelter W (2001). Pheromone cross-inhibition between *Staphylococcus aureus* and *Staphylococcus epidermidis*. Infect Immun.

[bib121] Otto M, Sussmuth R, Vuong C (1999). Inhibition of virulence factor expression in *Staphylococcus aureus* by the *Staphylococcus epidermidis agr* pheromone and derivatives. FEBS Lett.

[bib122] Pappas KM, Weingart CL, Winans SC (2004). Chemical communication in proteobacteria: biochemical and structural studies of signal synthases and receptors required for intercellular signalling. Mol Microbiol.

[bib123] Parashar V, Mirouze N, Dubnau DA (2011). Structural basis of response regulator dephosphorylation by Rap phosphatases. PLoS Biol.

[bib124] Parsek MR, Greenberg EP (2005). Sociomicrobiology: the connections between quorum sensing and biofilms. Trends Microbiol.

[bib125] Passador L, Cook JM, Gambello MJ (1993). Expression of *Pseudomonas aeruginosa* virulence genes requires cell-to-cell communication. Science.

[bib126] Pearson JP, Gray KM, Passador L (1994). Structure of the autoinducer required for expression of *Pseudomonas aeruginosa* virulence genes. P Natl Acad Sci USA.

[bib127] Pearson JP, Passador L, Iglewski BH (1995). A second N-acylhomoserine lactone signal produced by *Pseudomonas aeruginosa*. P Natl Acad Sci USA.

[bib128] Peng HL, Novick RP, Kreiswirth B (1988). Cloning, characterization, and sequencing of an accessory gene regulator (*agr*) in *Staphylococcus aureus*. J Bacteriol.

[bib129] Perego M. (1997). A peptide export-import control circuit modulating bacterial development regulates protein phosphatases of the phosphorelay. P Natl Acad Sci USA.

[bib130] Pereira CS, Thompson JA, Xavier KB (2013). AI-2-mediated signalling in bacteria. FEMS Microbiol Rev.

[bib131] Pesci EC, Pearson JP, Seed PC (1997). Regulation of *las* and *rhl* quorum sensing in *Pseudomonas aeruginosa*. J Bacteriol.

[bib132] Pestova EV, Havarstein LS, Morrison DA (1996). Regulation of competence for genetic transformation in *Streptococcus pneumoniae* by an auto-induced peptide pheromone and a two-component regulatory system. Mol Microbiol.

[bib133] Piper KR, Beck von Bodman S, Farrand SK (1993). Conjugation factor of *Agrobacterium tumefaciens* regulates Ti plasmid transfer by autoinduction. Nature.

[bib134] Plener L, Lorenz N, Reiger M (2015). The phosphorylation flow of the *Vibrio harveyi* quorum-sensing cascade determines levels of phenotypic heterogeneity in the population. J Bacteriol.

[bib135] Popat R, Crusz SA, Messina M (2012). Quorum-sensing and cheating in bacterial biofilms. Proc Biol Sci.

[bib136] Pottathil M, Lazazzera BA (2003). The extracellular Phr peptide-Rap phosphatase signaling circuit of *Bacillus subtilis*. Front Biosci.

[bib137] Rocha-Estrada J, Aceves-Diez AE, Guarneros G (2010). The RNPP family of quorum-sensing proteins in Gram-positive bacteria. Appl Microbiol Biot.

[bib138] Ryan RP, An SQ, Allan JH (2015). The DSF Family of Cell-Cell Signals: an expanding class of bacterial virulence regulators. PLoS Pathog.

[bib139] Sakoulas G, Eliopoulos GM, Moellering RC (2002). Accessory gene regulator (*agr*) locus in geographically diverse *Staphylococcus aureus* isolates with reduced susceptibility to vancomycin. Antimicrob Agents Ch.

[bib140] Sappington KJ, Dandekar AA, Oinuma K (2011). Reversible signal binding by the *Pseudomonas aeruginosa* quorum-sensing signal receptor LasR. MBio.

[bib141] Schaefer AL, Hanzelka BL, Eberhard A (1996a). Quorum sensing in *Vibrio fischeri*: probing autoinducer-LuxR interactions with autoinducer analogs. J Bacteriol.

[bib142] Schaefer AL, Val DL, Hanzelka BL (1996b). Generation of cell-to-cell signals in quorum sensing: acyl homoserine lactone synthase activity of a purified *Vibrio fischeri* LuxI protein. P Natl Acad Sci USA.

[bib143] Schuster M, Lostroh CP, Ogi T (2003). Identification, timing, and signal specificity of *Pseudomonas aeruginosa* quorum-controlled genes: a transcriptome analysis. J Bacteriol.

[bib144] Schuster M, Urbanowski ML, Greenberg EP (2004). Promoter specificity in *Pseudomonas aeruginosa* quorum sensing revealed by DNA binding of purified LasR. P Natl Acad Sci USA.

[bib145] Shadel GS, Young R, Baldwin TO (1990). Use of regulated cell lysis in a lethal genetic selection in *Escherichia coli*: identification of the autoinducer-binding region of the LuxR protein from *Vibrio fischeri* ATCC 7744. J Bacteriol.

[bib146] Shi K, Brown CK, Gu ZY (2005). Structure of peptide sex pheromone receptor PrgX and PrgX/pheromone complexes and regulation of conjugation in *Enterococcus faecalis*. P Natl Acad Sci USA.

[bib147] Simon M, Crane B, Crane A (2007). Two-Component Signaling Systems.

[bib148] Slamti L, Lereclus D (2002). A cell-cell signaling peptide activates the PlcR virulence regulon in bacteria of the *Bacillus cereus* group. EMBO J.

[bib149] Slamti L, Lereclus D (2005). Specificity and polymorphism of the PlcR-PapR quorum-sensing system in the *Bacillus cereus* group. J Bacteriol.

[bib150] Slock J, VanRiet D, Kolibachuk D (1990). Critical regions of the *Vibrio fischeri* LuxR protein defined by mutational analysis. J Bacteriol.

[bib151] Smith RS, Iglewski BH (2003). *Pseudomonas aeruginosa* quorum sensing as a potential antimicrobial target. J Clinical Invest.

[bib152] Stauff DL, Bassler BL (2011). Quorum sensing in *Chromobacterium violaceum*: DNA recognition and gene regulation by the CviR receptor. J Bacteriol.

[bib153] Stevens AM, Dolan KM, Greenberg EP (1994). Synergistic binding of the *Vibrio fischeri* LuxR transcriptional activator domain and RNA polymerase to the *lux* promoter region. P Natl Acad Sci USA.

[bib154] Stevens AM, Fujita N, Ishihama A (1999). Involvement of the RNA polymerase alpha-subunit C-terminal domain in LuxR-dependent activation of the *Vibrio fischeri* luminescence genes. J Bacteriol.

[bib155] Stevens AM, Greenberg EP (1997). Quorum sensing in *Vibrio fischeri*: essential elements for activation of the luminescence genes. J Bacteriol.

[bib156] Surette MG, Miller MB, Bassler BL (1999). Quorum sensing in *Escherichia coli*, *Salmonella typhimurium*, and *Vibrio harveyi*: a new family of genes responsible for autoinducer production. P Natl Acad Sci USA.

[bib157] Swem LR, Swem DL, O'Loughlin CT (2009). A quorum-sensing antagonist targets both membrane-bound and cytoplasmic receptors and controls bacterial pathogenicity. Mol Cell.

[bib158] Swem LR, Swem DL, Wingreen NS (2008). Deducing receptor signaling parameters from *in vivo* analysis: LuxN/AI-1 quorum sensing in *Vibrio harveyi*. Cell.

[bib159] Tiaden A, Spirig T, Hilbi H (2010). Bacterial gene regulation by alpha-hydroxyketone signaling. Trends Microbiol.

[bib160] Tomasz A (1965). Control of the competent state in Pneumococcus by a hormone-like cell product: an example for a new type of regulatory mechanism in bacteria. Nature.

[bib161] Travisano M, Velicer GJ (2004). Strategies of microbial cheater control. Trends Microbiol.

[bib162] Urbanowski ML, Lostroh CP, Greenberg EP (2004). Reversible acyl-homoserine lactone binding to purified *Vibrio fischeri* LuxR protein. J Bacteriol.

[bib163] Vannini A, Volpari C, Gargioli C (2002). The crystal structure of the quorum sensing protein TraR bound to its autoinducer and target DNA. EMBO J.

[bib164] Venturi V. (2006). Regulation of quorum sensing in *Pseudomonas*. FEMS Microbiol Rev.

[bib165] Vlamakis H, Aguilar C, Losick R (2008). Control of cell fate by the formation of an architecturally complex bacterial community. Gene Dev.

[bib166] Wagner VE, Gillis RJ, Iglewski BH (2004). Transcriptome analysis of quorum-sensing regulation and virulence factor expression in *Pseudomonas aeruginosa*. Vaccine.

[bib167] Waters CM, Bassler BL (2005). Quorum sensing: cell-to-cell communication in bacteria. Annu Rev Cell Dev B.

[bib168] Wei Q, Ma LZ (2013). Biofilm matrix and its regulation in *Pseudomonas aeruginosa*. Int J Mol Sci.

[bib169] Wei Y, Perez LJ, Ng WL (2011). Mechanism of *Vibrio cholerae* autoinducer-1 biosynthesis. ACS Chem Biol.

[bib170] Welsh MA, Blackwell HE (2016). Chemical genetics reveals environment-specific roles for quorum sensing circuits in *Pseudomonas aeruginosa*. Cell Chem Biol.

[bib171] West SA, Griffin AS, Gardner A (2006). Social evolution theory for microorganisms. Nat Rev Microbiol.

[bib172] Whitehead NA, Barnard AM, Slater H (2001). Quorum-sensing in Gram-negative bacteria. FEMS Microbiol Rev.

[bib173] Williams P, Camara M (2009). Quorum sensing and environmental adaptation in *Pseudomonas aeruginosa*: a tale of regulatory networks and multifunctional signal molecules. Curr Opin Microbiol.

[bib174] Williams P, Camara M, Hardman A (2000). Quorum sensing and the population-dependent control of virulence. Philos T Roy Soc B.

[bib175] Wintjens R, Rooman M (1996). Structural classification of HTH DNA-binding domains and protein-DNA interaction modes. J Mol Biol.

[bib176] Wright JS, Lyon GJ, George EA (2004). Hydrophobic interactions drive ligand-receptor recognition for activation and inhibition of staphylococcal quorum sensing. P Natl Acad Sci USA.

[bib177] Zhang L, Ji G (2004). Identification of a staphylococcal AgrB segment(s) responsible for group-specific processing of AgrD by gene swapping. J Bacteriol.

[bib178] Zhang RG, Pappas KM, Brace JL (2002). Structure of a bacterial quorum-sensing transcription factor complexed with pheromone and DNA. Nature.

[bib179] Zhu J, Beaber JW, More MI (1998). Analogs of the autoinducer 3-oxooctanoyl-homoserine lactone strongly inhibit activity of the TraR protein of *Agrobacterium tumefaciens*. J Bacteriol.

[bib180] Zhu J, Miller MB, Vance RE (2002). Quorum-sensing regulators control virulence gene expression in *Vibrio cholerae*. P Natl Acad Sci USA.

[bib181] Zhu J, Winans SC (1999). Autoinducer binding by the quorum-sensing regulator TraR increases affinity for target promoters *in vitro* and decreases TraR turnover rates in whole cells. P Natl Acad Sci USA.

[bib182] Zhu J, Winans SC (2001). The quorum-sensing transcriptional regulator TraR requires its cognate signaling ligand for protein folding, protease resistance, and dimerization. P Natl Acad Sci USA.

[bib183] Zou Y, Nair SK (2009). Molecular basis for the recognition of structurally distinct autoinducer mimics by the *Pseudomonas aeruginosa* LasR quorum-sensing signaling receptor. Chem Biol.

[bib184] Zouhir S, Perchat S, Nicaise M (2013). Peptide-binding dependent conformational changes regulate the transcriptional activity of the quorum-sensor NprR. Nucleic Acids Res.

